# Factors associated with postoperative dysphagia in older adults with hip fracture: a scoping review

**DOI:** 10.3389/fsurg.2026.1769151

**Published:** 2026-02-04

**Authors:** Ganying Huang, Yitao Zhou, Huijie Yang, Tao Zhou, Yangbin Zhou, Qiuhua Sun

**Affiliations:** 1 The Fourth School of Clinical Medicine, Zhejiang Chinese Medical University (Hangzhou First People’s Hospital), Hangzhou, Zhejiang, China; 2 School of Nursing, Zhejiang Chinese Medical University, Hangzhou, China; 3 Emergency Department, Hangzhou First People's Hospital, Hangzhou, China

**Keywords:** hip fracture surgery, influencing factors, older adults, postoperative dysphagia, scoping review

## Abstract

**Background:**

Postoperative dysphagia is increasingly recognized as a common but underdiagnosed complication in older adults undergoing hip fracture surgery, and is associated with adverse outcomes such as aspiration pneumonia, malnutrition, prolonged hospitalization, and delayed rehabilitation. However, evidence regarding its reported incidence and influencing factors remains fragmented and heterogeneous.

**Objective:**

This scoping review aimed to map the existing evidence on the incidence, assessment methods, and factors associated with postoperative dysphagia in older adults undergoing hip fracture surgery, and to identify knowledge gaps relevant to perioperative management and rehabilitation.

**Design:**

This study was designed as a scoping review to map the existing evidence on postoperative dysphagia in older adults undergoing hip fracture surgery. The review was conducted in accordance with the Arksey and O'Malley framework and reported following the PRISMA-ScR guidelines.

**Methods:**

A comprehensive search was performed across nine English and Chinese databases from inception to June 2025. Studies invovling patients aged ≥ 60 years undergoing hip fracture surgery were included.Risk of bias assessment and descriptive quantitative synthesis were applied to characterize the heterogeneity of the evidence, rather than to generate definitive estimates.

**Results:**

Eleven studies were included. Six types of swallowing assessment methods were identified, ranging from bedside screening to videofluoroscopic swallowing studies. The reported incidence of postoperative dysphagia varied widely across studies. A descriptive quantitative synthesis yielded a pooled prevalence of 39% (95%CI:0.26–53); however, this estimate should be interpreted with caution due to extreme statistical heterogeneity (*I*^2^ = 99%) and variations in assessment timing. Influencing factors were grouped into patient-related, surgery-related, and sarcopenia-related domains. Evidence linking sarcopenia to persistent postoperative dysphagia was limited.

**Conclusions:**

Postoperative dysphagia in older adults with hip fracture is a multifactorial condition with a widely varying and inconsistently reported incidence. Differences in assessment methods and timing contribute substantially to heterogeneity across studies. Future research should focus on longitudinal recovery trajectories and the role of sarcopenia to inform targeted perioperative and rehabilitation interventions.

**Systematic Review Registration:**

https://doi.org/10.17605/OSF.IO/4NR9H.

## Background

With the intensification of global population aging, the incidence of fractures among older adults has risen markedly owing to factors such as osteoporosis, falls, and degenerative changes (1). Improvements in the quality of life and advances in medical technology have led to a steady increase in the number of orthopedic surgeries performed on older patients. However, age-related physiological degeneration often results in presbyphagia; following stressful events such as surgery, swallowing function may decline or even be lost because of intraoperative tissue injury, anesthetic effects, and impaired neuromuscular function (2, 3). Recent studies have reported that the prevalence of postoperative dysphagia ranges from 5.3% to 76.9% after hip fracture surgery (4–6), 1.0% to 83.3% after spinal surgery (7–10), and 6.4% to 14.2% after other orthopedic procedures (11). Such wide variations in prevalence may be attributed to differences in diagnostic criteria for dysphagia and sample sizes across studies.

Dysphagia in older adults is associated with stroke, neurodegenerative diseases, and head and neck cancer. With growing research on sarcopenic dysphagia, sarcopenia has also been recognized as a risk factor. Dysphagia can lead to adverse outcomes such as dehydration, aspiration pneumonia, and malnutrition (12–14); prolong hospital stay (15); impair postoperative recovery and quality of life; and increase the burden on families and society, thus becoming an escalating global socioeconomic challenge. While the epidemiology and harmful consequences of postoperative dysphagia in older adults undergoing hip fracture surgery are widely recognized, recent findings suggest that patients with hip fractures exhibit a longitudinal trajectory of swallowing dysfunction after surgery (16). However, large-scale studies on its prevalence and associated factors are scarce. Therefore, this study adopted the scoping review framework proposed by Arksey and O'Malley to systematically examine the prevalence of and factors influencing postoperative dysphagia in older adults undergoing hip fracture surgery, aiming to provide evidence for early identification and individualized interventions.

## Methods

### Protocol registration

The review was reported as per the Preferred Reporting Items for Systematic Reviews and Meta-Analyses extension for Scoping Reviews (PRISMA-ScR) guidelines (17). The protocol of the review was registered in OSF September 2025 (https://doi.org/10.17605/OSF.IO/4NR9H).

This review addressed the following research questions: (1) What is the prevalence of postoperative dysphagia in older adults undergoing hip fracture surgery? (2) What are the factors associated with postoperative dysphagia in this population?.

### Eligibility criteria

Studies were eligible for inclusion if they met the following criteria: (1) participants were adults aged ≥60 years who underwent hip fracture surgery; (2) postoperative dysphagia was reported as an outcome; (3) observational study designs, including cross-sectional, cohort, or case–control studies; and (4) articles published in English or Chinese.

Although broader orthopedic procedures were initially considered during the search phase, the final included studies overwhelmingly focused on hip fracture surgery. To improve internal consistency and clinical relevance, the scope of the review was refined at the full-text assessment stage.

### Informaion sources and search strategy

A comprehensive literature search was conducted across nine electronic databases, including PubMed, Web of Science, MEDLINE, EMBASE, CINAHL, Cochrane Library, China National Knowledge Infrastructure (CNKI), Wanfang Data, VIP Database, and Chinese Biomedical Literature Database, covering all records from database inception to June 26, 2025. The following English search terms were used: “Aged/Elderly/Old,” “Orthopedic Procedures/Orthopedic Procedure*/Orthopedic Surger*/Orthopedic Rehabilitation Surger*/Orthopedic Surgical Procedure*,” and “Deglutition Disorders/Deglutition Disorder*/Dysphagia/Swallowing Disorder*/Oropharyngeal Dysphagia/Esophageal Dysphagia.”

The detailed search strategy was as follows: English databases (e.g., PubMed). #1

"Aged"\[MeSH Terms] OR "Elderly"\[Title/Abstract] OR "Old"\[Title/Abstract]

#2"Orthopedic Procedures"\[MeSH Terms] OR “Orthopedic Procedure*"[Title/Abstract] OR "Orthopedic Surger*"[Title/Abstract] OR "Orthopedic Rehabilitation Surger*"[Title/Abstract] OR “Orthopedic Surgical Procedure*"[Title/Abstract]

#3"Deglutition Disorders"[MeSH Terms] OR “Deglutition Disorder*"[Title/Abstract] OR “Dysphagia"[Title/Abstract] OR “Swallowing Disorder*"[Title/Abstract] OR “Oropharyngeal Dysphagia"[Title/Abstract] OR "Esophageal Dysphagia"[Title/Abstract]

#4 #1 AND #2 AND #3.

### Risk of bias assessment

To provide contextual information on study quality, the risk of bias of the included studies was assessed using a tool for non-randomized studies. This assessment was conducted to support interpretation of the evidence map rather than to exclude studies or to weight pooled estimates.

### Study selection and data charting

All retrieved records were imported into EndNote for deduplication. Two reviewers independently screened titles and abstracts, followed by full-text assessment of potentially eligible articles. Any disagreements were resolved through discussion or consultation with a third reviewer.

Data were independently charted by two reviewers using a standardized data charting form. The charted information included author, year of publication, country, study design, participant characteristics, swallowing assessment methods, reported prevalence of postoperative dysphagia, and associated factors.

### Data synthesis

Data were synthesized descriptively to summarize the range of reported prevalence estimates and associated factors. Where appropriate, a descriptive quantitative synthesis using random-effects modeling was conducted to illustrate the variability and heterogeneity of reported prevalence across studies, rather than to derive a definitive pooled estimate. Statistical heterogeneity was assessed using the *I*^2^ statistic.

## Results

### Study selection

**Figure 1:**
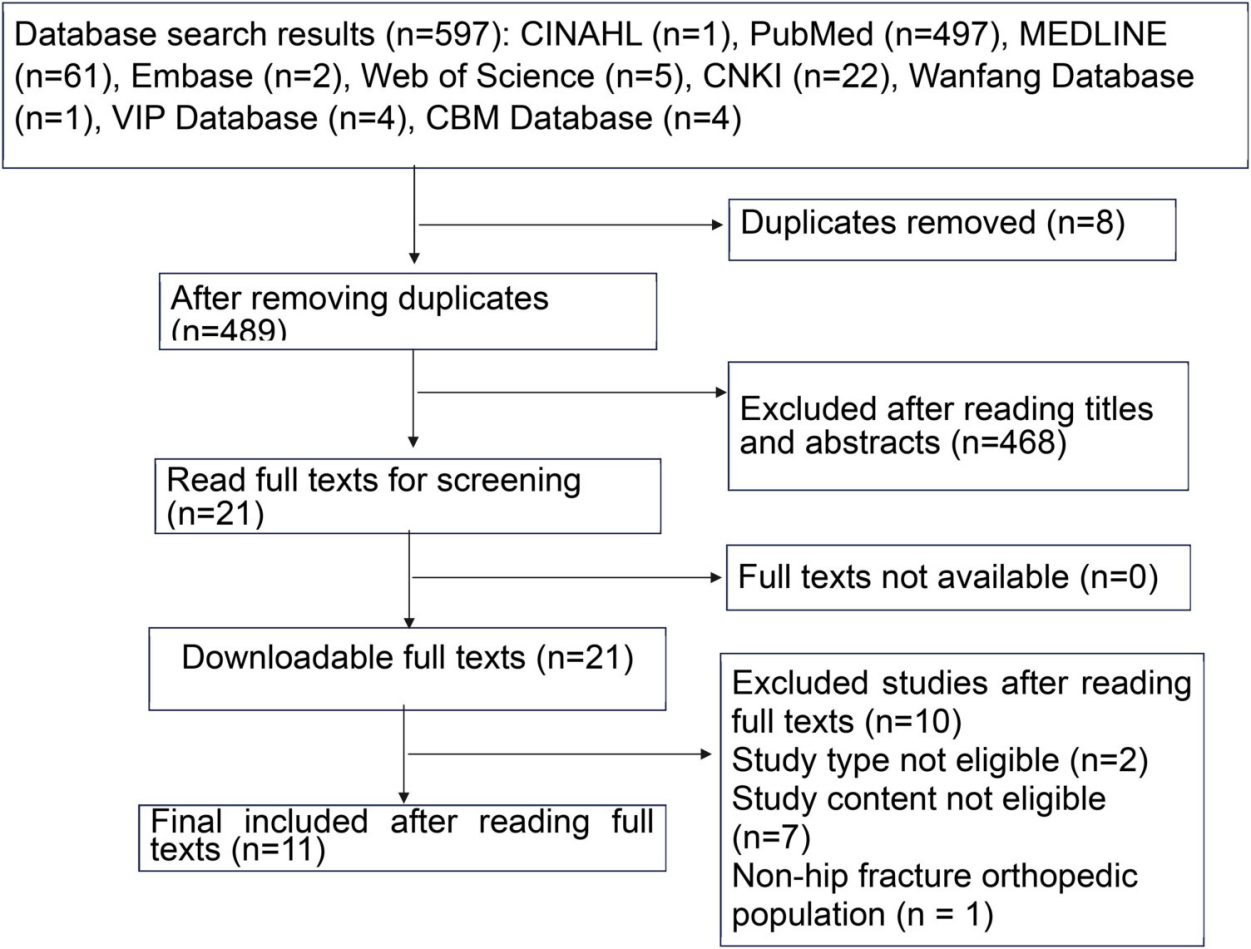
PRISMA flow diagram of the study selection process. The diagram illustrates the systematic identification, screening, and inclusion of 11 studies investigating postoperative dysphagia in older adults undergoing hip fracture surgery.

A total of 597 records were identified. After removing 8 duplicates, 489 articles remained. After title and abstract screening, 468 articles that did not meet the inclusion criteria were excluded. After full-text review, 10 articles were excluded because of inappropriate study design, irrelevant content, or a focus on non-hip fracture orthopedic surgery. Finally, 11 studies were included in this review. The study selection process is illustrated in Figure 1.

### Characteristics of the included studies

Eleven studies from seven countries were included: Australia (*n* = 2), the United States (*n* = 2), Korea (*n* = 1), Denmark (*n* = 1), Japan (*n* = 3), Spain (*n* = 1), and the Netherlands (*n* = 1). All included studies focused on older adults undergoing hip fracture surgery. Table 1 summarizes the basic characteristics of the studies.

**Table 1:** Characteristics of the included studies in older adults undergoing hip fracture surgery (*n* = 11).

No.	Author (Year)	Country	Study Design	Sample Size (male/female)	Age (years)	Exclusion of preoperative dysphagia	Assessment Tool	Assessment Time	Prevalence (%)	Associated Factors
1	Love et al. (20)	Australia	Prospective	181 (54/127)	83.8 ± 8.1	Yes	Clinical swallowing assessment (e.g., medical history, oral muscle exam, voice assessment, Australian standardized diet test)	72 h post-op	61 (33.7%)	Age, pre-admission residence, neurological, respiratory, cardiac, ENT comorbidities, number of comorbidities, anesthesia type, ICU admission, postoperative delirium
2	Meals et al. (5)	USA	Retrospective	52 (16/36)	81 (65–98)	Yes	Bedside swallowing evaluation (mental status, oxygen requirement, oral mucosa, swallowing, post-swallow events, vital signs, subjective swallowing symptoms)	72 h post-op	22 (42.3%)	Age, sex, ASA classification
3	Beric et al. (19)	Australia	Prospective	103 (25/78)	81.5 ± 8.1 (65–94)	Not reported	Bedside swallowing evaluation	Within 72 h post-op	56 (54.4%)	Sex, residence, neurological/cardiac/ENT/dementia/UTI/respiratory/malnutrition/dehydration comorbidities, polypharmacy, previous hip fracture, LOS, residence type, family support, surgery type, fracture type, anesthesia type, ICU admission, postoperative confusion, dyspnea, NG tube requirement
4	Byun et al. (4)	Korea	Retrospective	546 (153/393)	80.3 ± 7.0	Not reported	VFSS	At admission	29 (5.3%)	Serum albumin < 3.5 g/dL
5	Madsen et al. (6)	Denmark	Cross-sectional	78 (24/54)	81.4 ± 7.8	Not reported	Standardized Volume–Viscosity Swallow Test	Within 72 h post-op	60 (76.9%)	Pre-admission residence in nursing home, low habitual NMS, absence of cardiac comorbidities significantly associated with swallowing/feeding difficulties
6	Nagano et al. (16)	Japan	Retrospective	89 (0/89)	85.9 ± 6.5	Yes	FOIS	Post-op, day 7, at discharge	Overall: 27 (29.3%)	SMI, handgrip strength, GNRI, pre-injury physical score, ASA classification
									Day 7: 11 (12.3%); Discharge: 12 (13.5%);	
7	Mateos-Nozal et al. (24)	Spain	Prospective	320 (85/235)	86.2 ± 6.1	Not reported	Volume–Viscosity Swallow Test	Within 72 h post-op	176 (55%)	Age, sex, residence, Barthel Index, FAC, GDS, MNA-SF, BMI, dementia, vascular disease, Parkinson’s, stroke, head/neck cancer, malnutrition, polypharmacy, fracture type, in-hospital complications, delirium, transfusion, LOS
8	Wijnen et al. (21)	Netherlands	Retrospective	92 (20/72)	83.5 ± 7.4	Yes	Standardized swallowing assessment	Post-op	27 (29.3%)	Age, sex, residence, use of walking aid, previous dysphagia, SLP consultation, diet modification, cerebrovascular disease, Parkinson’s/dementia, Charlson Comorbidity Index
9	Boyapati et al. (18)	USA	Retrospective	617 (NR/NR)	≥65	Yes	Medical records	Post-op	347/617 (56.2%); Chronic 46.4%; Acute 53.6%	Perioperative dysphagia events, acute dysphagia associated with mortality
10	Kiyomiya et al. (22)	Japan	Prospective	21 (0/21)	≥65	Not reported	Water Swallow Test	48 h, 2 weeks post-op	11 (52.4%)	Age, weight, BMI, FILS, GNRI, BI, CRP, GH length
11	Tamura et al. (23)	Japan	Retrospective	88,809 (NR)	>60	Not reported	WST, RSST, Simple Swallow Provocation Test, VFSS, or clinical symptoms	Post-op	1822 (2.1%)	Age, sex, BMI, fracture type, surgical variables, frailty score, in-hospital procedures, medications

VFSS, videofluorographic swallowing study; EAT- 10, the eating assessment tool (EAT-10); WST, water swallowing test; ASA, American society of anesthesiologists classification; FAC, functional ambulation category; GDS, geriatric depression scale; MNA-SF, mini nutritional assessment - short form; BMI, body mass index; FILS, food Intake level scale; GNRI, geriatric nutritional risk index; BI, barthel index, CRP, C-reactive protein.

### Screening and assessment tools for dysphagia

The methods used to screen for or assess dysphagia varied among the 11 studies. One study did not describe the screening or assessment methods (18). Three studies used bedside screening (19, 20). Six studies used quantitative tests, including the Volume–Viscosity Swallow Test, Water Swallow Test, and standardized swallowing assessments (6, 11, 21–24). One study employed a scoring tool based on oral intake status (16), another employed a questionnaire (11), and another conducted a Videofluorographic Swallowing Study (VFSS) (4).

### Assessors and timing of dysphagia evaluation

In more than half of the included studies, dysphagia was assessed by experts or healthcare professionals under expert supervision. All studies performed postoperative evaluations. Four studies did not specify the exact timing of assessment and only indicated that evaluation was conducted after admission (4, 18, 21, 23).

### Quality assessment

**Figure 2:**
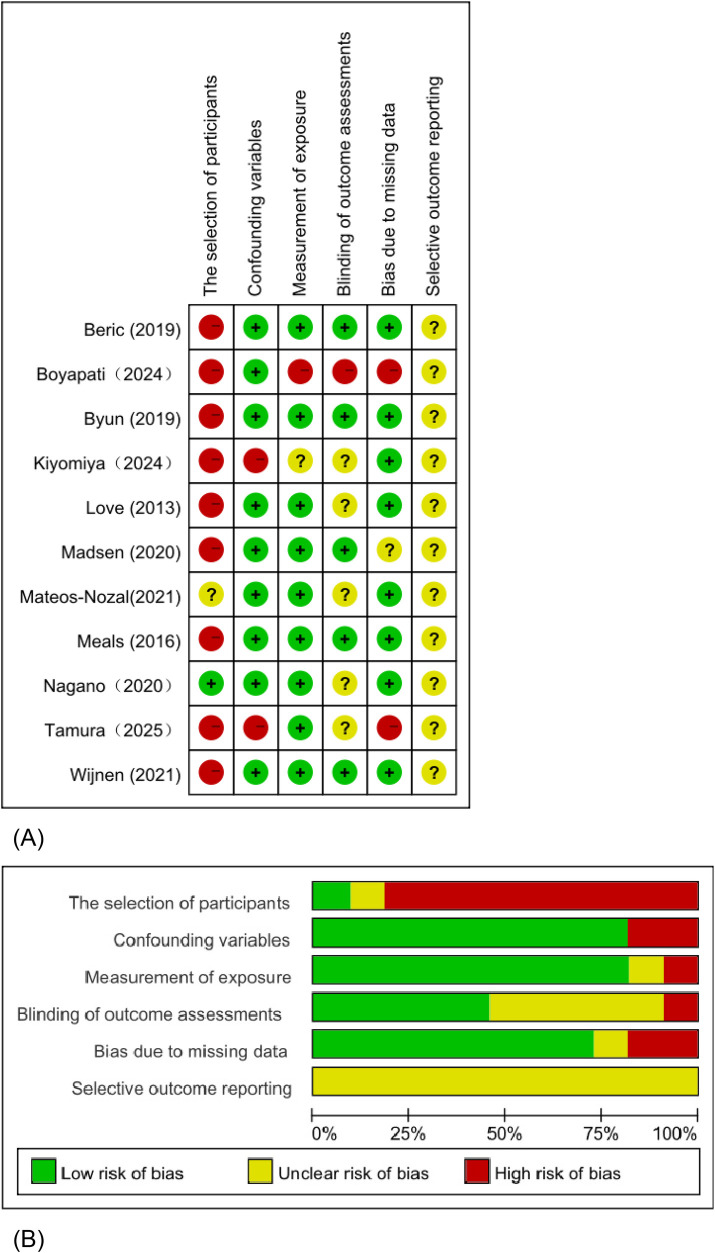
Risk of bias assessment of the included studies. **(A)** Traffic light plot showing the risk of bias judgments for each study across six methodological domains: selection of participants, confounding variables, measurement of exposure, blinding of outcome assessments, bias due to missing data, and selective outcome reporting. **(B)** Summary plot presenting the proportion of studies rated as low, unclear, or high risk of bias within each domain. Green indicates low risk of bias, yellow indicates unclear risk of bias, and red indicates high risk of bias.

The results of the quality assessment are shown in Figure 2. Overall, a high risk of bias was observed in the domain of participant selection. Several studies showed unclear or high risk of bias regarding control of confounding variables and blinding of outcome assessment, while the risk of bias related to outcome measurement and missing data was generally low.

### Reported incidence of postoperative dysphagia in older adults undergoing hip fracture surgery

**Figure 3:**
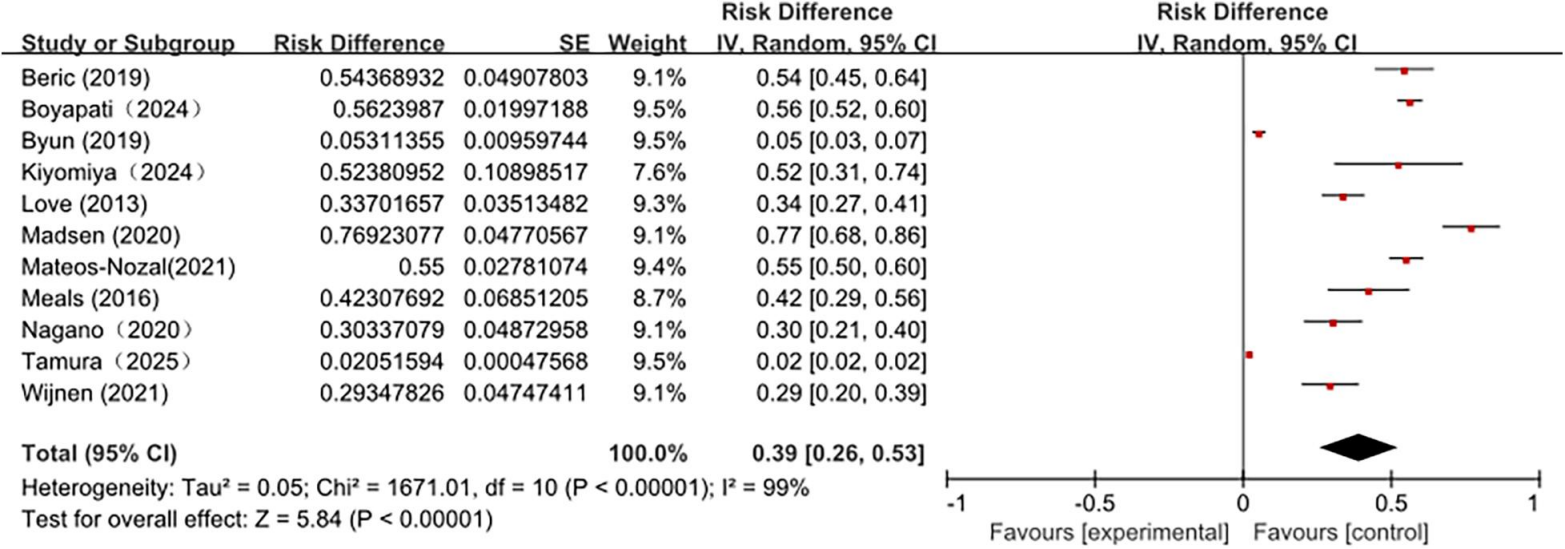
Forest plot showing the pooled prevalence of postoperative dysphagia among older adults undergoing hip fracture surgery. A descriptive quantitative synthesis using a random-effects model was performed. The pooled prevalence was 39% (95% CI: 26–53), with extreme heterogeneity across studies (*I*^2^ = 99%). This pooled estimate should be interpreted as a descriptive indicator reflecting substantial variability in assessment tools, timing of evaluation, and study populations.

As shown in Figure 3, a descriptive quantitative synthesis using random-effects modeling indicated that the pooled prevalence of postoperative dysphagia among older adults undergoing hip fracture surgery was 39% (95% CI: 26–53). The forest plot demonstrated extreme heterogeneity (*I*^2^ = 99%), reflecting substantial variability across studies. The heterogeneity is likely attributable to differences in the study design (mostly observational studies), patient populations, assessment tools, timing of evaluation, and sample sizes. In particular, the variability in dysphagia screening and diagnostic methods appeared to be a major factor. Similar heterogeneity has also been reported in dysphagia screening studies conducted in nursing home settings (25).Therefore, the pooled prevalence estimate should be interpreted with caution and viewed as a broad descriptive indicator rather than a definitive epidemiological estimate.

### Factors associated with postoperative dysphagia in older adults undergoing hip fracture surgery

All 11 studies reported the risk factors for postoperative dysphagia (Table 1), Frequently reported patient-related factors included advanced age (*n* = 8) (4, 6, 16, 19–21, 24), postoperative delirium or altered consciousness (*n* = 4) (6, 19–21), pre-admission living environment (*n* = 4) (19–21, 24), female sex (*n* = 1) (19), comorbid neurological or respiratory conditions (*n* = 5) (6, 19–21, 24), preoperative status according to the American Society of Anesthesiologists (ASA) classification (*n* = 2) (5, 16), longer time from admission to surgery (*n* = 2) (6, 23), sarcopenia (*n* = 2) (16, 22), and pre-admission mobility (*n* = 1) (24). Adverse outcomes associated with post-operative dysphagia were also reported. Two studies examined its impact on postoperative pneumonia and identified dysphagia as a significant risk factor (4, 5), while one study highlighted its association with increased mortality (23).

## Discussion

### Prevalence of postoperative dysphagia in older adults undergoing hip fracture surgery: variability related to assessment tools

In this scoping review, most included studies focused on older adulets undergoing hip fracture surgery. A descriptive quantitative synthesis indicated a pooled prevalence of postoperative dysphagia 39% (CI: 26–53), however, this estimate was accompanied by extreme heterogeneity. This wide variability suggests that the reported prevalence should be interpreted as a descriptive indicator rather than a definitive epidemiological estimate. Differences in sample sizes, swallowing assessment tools, and the absence of preoperative dysphagia assessment likely contributed to this heterogeneity.

Among these factors, the choice of the assessment tool appeared to be a crucial factor. Dysphagia was evaluated using objective methods, such as dietary intake information, standardized functional assessments, and Videofluoroscopic Swallowing Study (VFSS), as well as subjective methods, such as self-reported questionnaires. VFSS typically yielded lower prevalence estimates than other methods; however, it requires specialized personnel. Whereas, subjective assessments may overestimate the prevalence relative to objective evaluations. The assessors included both nursing staff and speech-language pathologists. For clinical applicability, it is essential to consider that nurses form the frontline workforce. Therefore, the selection of appropriate tools for nurse-led assessments and risk screening is particularly important. Future research should focus on developing more sensitive tools tailored for older orthopedic or hospitalized patients or combining multiple tools to improve the accuracy of dysphagia screening and assessment in this population.

### Variation in timing of assessment as an important contributor heterogeneity

The timing of dysphagia screening and assessment varied across studies, ranging from the immediate postoperative period to 48 or 72 h after surgery, postoperative day 7, two weeks after surgery, at hospital discharge, or upon transfer to rehabilitation facilities. This variability likely contributed substantially to the observed heterogeneity in reported prevalence.

Despite this variability, several studies suggested a potential postoperative trajectory of swallowing recovery. For example, Nagano et al. (16) reported that among older women undergoing hip fracture surgery, the prevalence of dysphagia was 12.3% on postoperative day 7 and 13.5% at discharge. Similarly, Kiyomiya et al. (22) observed a gradual decline in dysphagia prevalence over time. These findings suggest that swallowing recovery may follow heterogeneous trajectories across different postoperative time points, possibly reflecting distinct underlying mechanisms. However, current evidence remains limited, and no formal trajectory analyses have been conducted. Therefore, longitudinal studies examining the temporal course of postoperative dysphagia are clinically warranted.

### Factors influencing postoperative dysphagia in older adults undergoing hip fracture surgery

This review aimed to map the reported prevalence of postoperative dysphagia following hip fracture surgery in older adults and to summarize factors reported to be associated with this condition. Several patient-related factors were reported to be associated with an increased risk of postoperative dysphagia, including advanced age, history of neurological or respiratory comorbidities, postoperative delirium, and indicators of poor nutritional status such as hypoalbuminemia. Evidence regarding the roles of sex and body mass index (BMI) was inconsistent, although one study reported that women were more likely to develop dysphagia. Furthermore, preoperative residence in a nursing facility may reflect underlying frailty and poor nutritional status in this population. Frailty-related health status was identified as a relevant factor in several studies, although poor oral health was not explicitly reported as a risk factor (26, 27). No significant association was observed for socioeconomic and educational status. While some studies have demonstrated strong associations between cognitive impairment, sensory deficits, and dysphagia (28), these variables were not reported in the studies included in this review, warranting further investigation.

Surgery-related risk factors reported in the included studies primarily involved prolonged. Waiting time before surgery. Anesthesia-related variables were not explicitly addressed in the included studies. In contrast, other studies reported associations between dysphagia and factors such as intubation time, cuff pressure, tube material or size, recurrent laryngeal nerve injury, and duration of mechanical ventilation (29–31). D'Haese et al. demonstrated that replacing endotracheal tubes with tapered polyurethane-cuffed tubes could reduce the risk of intraoperative aspiration (28). Shimizu et al. also identified associations between intubation method, operative time, and postoperative dysphagia. Wang et al. reported that difficult intubation, higher anesthetic risk classification, and perioperative airway management issues were predictive factors for postoperative dysphagia (32). Furthermore, Boyapati et al. noted that perioperative dysphagia events and acute-onset dysphagia were associated with increased mortality, whereas, Meals et al. identified dysphagia as a predictor of postoperative pneumonia. These findings underscore the potential clinical importance of early identification and appropriate management of postoperative dysphagia, while avoiding overinterpretation beyond the available evidence.

### Sarcopenia as an emerging but limited factor

Sarcopenia has emerged as an increasingly discussed but still limited factor in relation to postoperative dysphagia. Wakabayashi et al.reported that reduced skeletal muscle mass was highly prevalent in patients with dysphagia after cardiovascular surgery (33). Kuzuya et al. suggested that anesthetic agents, including atracurium, cisatracurium, and aminosteroidal neuromuscular blockers such as vecuronium and pancuronium, may induce sarcopenia (34). The Japanese scholar Akio Shimizu described delayed dysphagia, defined as swallowing dysfunction occurring seven days after an acute stroke, and highlighted its close association with low muscle strength and mass (35). This mechanism may be parallel to that observed in postoperative orthopedic patients and is likely related to reduced physical activity (36). Similarly, Bautmans et al. demonstrated that higher levels of surgery-induced inflammation were significantly associated with impaired muscle function and increased fatigue, observed within days after surgery and persisting beyond one month (37).

Collectively, these findings suggest that a postoperative decline in muscle mass and function may play a role in the development of dysphagia or delayed-onset dysphagia in older patients. However, only two of the studies included in this review directly examined sarcopenia as a contributing factor, and the current evidence remains limited. Further well-designed studies are therefore needed to clarify the role of sarcopenia in postoperative dysphagia among older adults undergoing hip fracture surgery.

## Summary

This scoping review aimed to describe and synthesize the reported prevalence of postoperative dysphagia and the associated risk factors among older adults undergoing hip fracture surgery. Although a broader range of orthopedic procedures was initially considered, the scope was refined to focus on hip fractures to improve clinical consistency and methodological coherence. The reported pooled prevalence of 39% was derived from studies with limited sample sizes and predominantly observational, heterogeneous designs, which contributed to substantial variability; thus, this value should be interpreted as a broad descriptive indicator rather than a definitive estimate. This review identified several factors linked to postoperative dysphagia, including advanced age, preoperative health status, malnutrition, and pre-admission living environment. Furthermore, preliminary evidence suggests that sarcopenia during the rehabilitation phase may be associated with the risk of dysphagia. These findings highlight the need for further longitudinal research to delineate the trajectory of postoperative dysphagia and evaluate the efficacy of targeted rehabilitation interventions following hip fracture surgery.

### Limitations

This review has several limitations. First, although a broad range of orthopedic procedures was initially considered, the final scope was restricted to hip fracture surgery to improve population homogeneity. As a result, the findings may not be generalizable to other orthopedic populations, such as patients undergoing cervical spine surgery, which involves distinct neuroanatomical risks for dysphagia. Second, the pooled prevalence of postoperative dysphagia (39%) was characterized by extreme heterogeneity(*I*^2^ = 99%), reflecting substantial variability in assessment methods, timing of evaluation, and study designs. Accordingly, this estimate should be interpreted as a broad descriptive indicator rather than a definitive epidemiological measure. Third, evidence regarding certain risk factors, particularly sarcopenia, remains preliminary due to the limited number of studies directly addressing these variables. Finally, the restriction to English- and Chinese-language publications may have led to the exclusion of relevant studies published in other languages.

## Data Availability

The original contributions presented in the study are included in the article/Supplementary Material, further inquiries can be directed to the corresponding author.
